# One pot synthesis of silver nanoparticles using a cyclodextrin containing polymer as reductant and stabilizer

**DOI:** 10.3762/bjnano.5.44

**Published:** 2014-03-31

**Authors:** Arkadius Maciollek, Helmut Ritter

**Affiliations:** 1 Institute of Organic Chemistry and Macromolecular Chemistry, Heinrich-Heine-University Duesseldorf, Universitaetsstraße 1, 40225 Duesseldorf, Germany

**Keywords:** cyclodextrin, polymer, silver nanoparticles, surface plasmon resonance

## Abstract

A facile and one pot synthesis of silver nanoparticles with narrow size distributions using silver nitrate and a copolymer **1** from *N*-isopropylacrylamide (NIPAM) and mono-(1*H*-triazolylmethyl)-2-methylacryl-β-cyclodextrin acting as reductant and stabilizer without using any additional reducing agent is reported. The reduction was carried out in aqueous solution under pH neutral conditions at room temperature. The results of dynamic light scattering analysis and transmission electron microscopy show adjustable particle sizes from 30–100 nm, due to variation of silver nitrate concentration, the polymeric reducing and stabilisation agent concentration or reaction time. The spherical structure of the silver nanoparticles has been confirmed by UV–vis spectroscopy and transmission electron microscopy. The optical properties of the nanoparticles have also been characterized by fluorescence spectroscopy. The formed spherical particles are stable in aqueous medium at room temperature over a period of several weeks. Furthermore the changes in the optical properties of the nanoparticles due to thermo induced volume phase transition behavior of the thermoresponsive cyclodextrin containing polymer **1** have been characterized by UV–vis spectroscopy.

## Introduction

Recently the interest in noble metal nano scaled particles increased significantly. Due to their unique physicochemical and microbacterial properties silver nanoparticles (AgNP) are one of the most studied in the field of nanotechnology and have a broad range of applications including optics, catalysis and biomedical science [1–4]. Many strategies have been established in the formation of metal nanoparticles, for example chemical or electrochemical reduction, irradiation or thermal decomposoion [5–8].

Most of these methods require environmental and biological risky reducing agents and solvents such as sodium borohydride, hydrazine or dimethylformamide. Consequently, the interest in a green nanoparticle synthesis using natural reducing agents like saccharides or cyclodextrin (CD) in environmentally benign solvents increased [9–13]. In the synthesis of metal nanoparticles the use of polymers as external steric or electrosteric stabilizer is an established method to control the particle growth, limit oxidation and stabilize the nanoparticle dispersion [5,14–16]. However, there are only a few examples for polymers described which act simultaneously as reducing and stabilization agent in aqueous medium. Typical examples are polyacrylamide, poly(sodium acrylate), polyethylene glycol, chitosan or soluble starch [11,17–19]. However, up to now polymers bearing CD attached have not been used for the preparation of AgNP’s. Thus, the scope of our present investigation was a new and facile synthesis of stable silver nanoparticles in aqueous medium. In this present work stable spherical silver nanoparticles are prepared in water using a copolymer **1** from *N*-isopropylacrylamide and mono-(1*H*-triazolylmethyl)-2-methylacryl-β-cyclodextrin [20] as reduction and steric stabilization agent without any additional reduction agent or energy source. Silver nitrate has been reduced under pH neutral conditions at room temperature. In this green one pot synthesis neither commonly used toxic reduction agent nor additional energy sources are needed. Through variation of the silver nitrate or polymer concentration silver nanoparticles of different sizes can be prepared.

## Results and Discussion

**Scheme 1:**
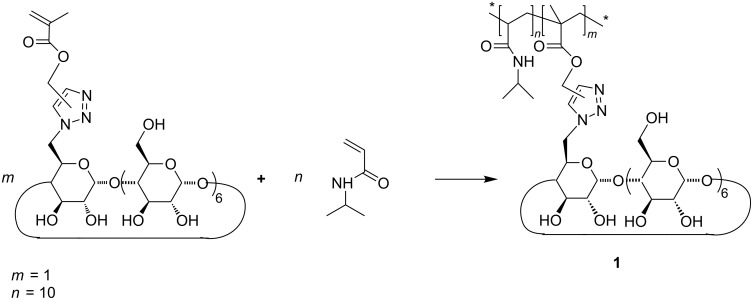
Synthesis of of β-cyclodextrin containing polymer **1**.

Long time stable silver nanoparticles were synthesized by the reaction of silver nitrate with an pH neutral aqueous solution of β-cyclodextrin containing polymer **1** at room temperaure. The copolymer 1 was prepared from *N*-isopropylacrylamide and mono-(1*H*-triazolylmethyl)-2-methylacryl-β-cyclodextrin via free radical polymeraization (Scheme 1). The molar weight *M*_n_ = 2·10^3^ g/mol was measured by aqueos SEC-MALS (D = 3.2). Through variation of the silver nitrate or polymer concentration silver nanoparticles of different sizes can be prepared. To avoid reduction of the silver salt by sodium ascorbate the microwave assisted click-type triazol-CD-monomer synthesis was carried out in the absence of copper(II) sulfate pentahydrate and sodium ascorbate compared to the origin literature, yielding in a mixture of 1,4- and 1,5-triazole regioisomers [21].

**Figure 1:**
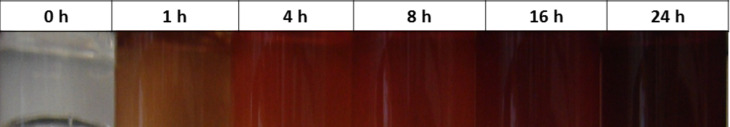
Photographs of silver nanoparticles **2** after different stirring times.

The color of the polymer solution changed during the reaction from colorless, over orange to brown (Figure 1) confirming the formation of the silver nanoparticles **2**.

**Figure 2:**
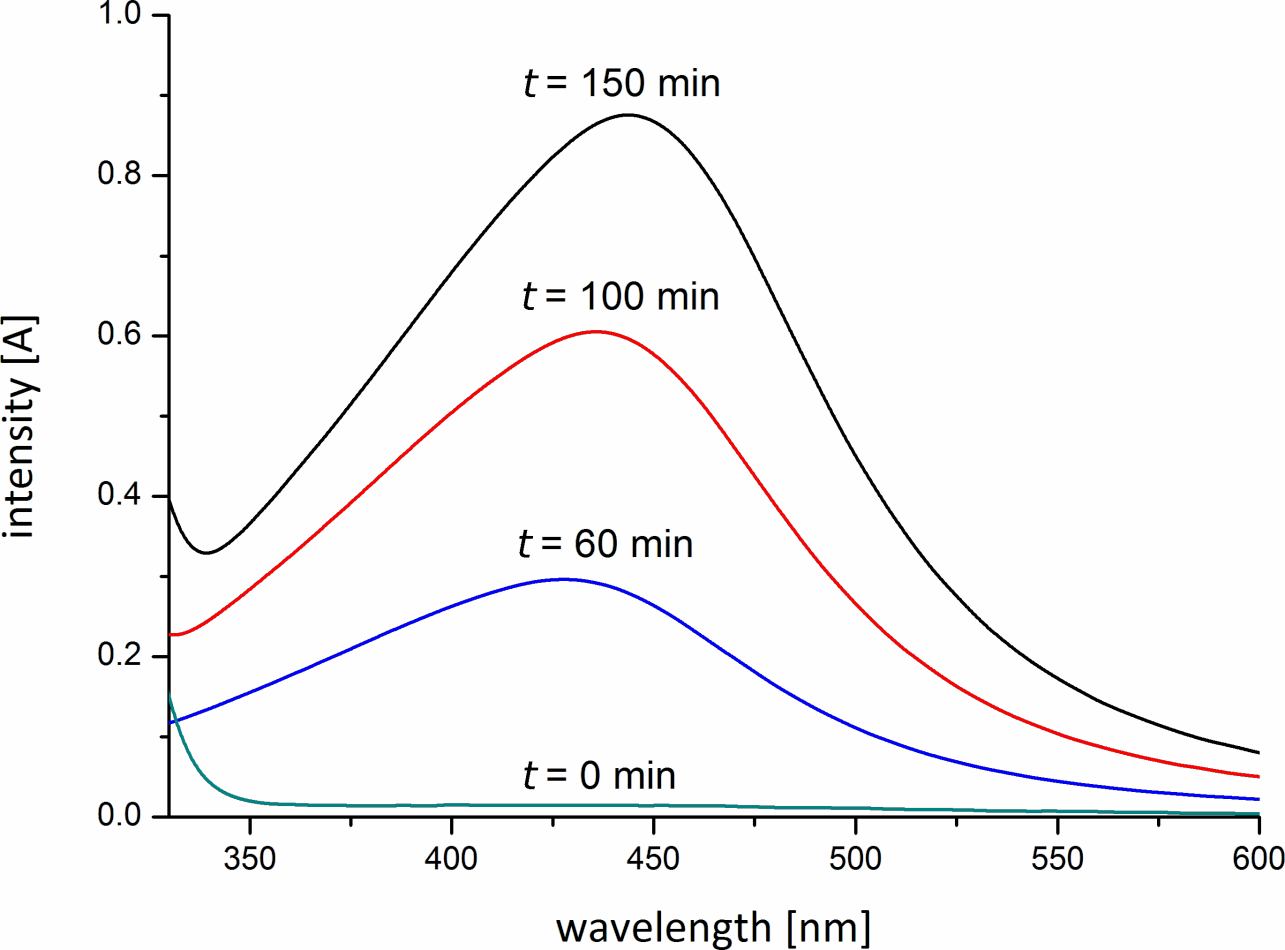
UV–vis absorption spectra of silver nanoparticles **2a** after different stirring times.

This optical activity is a result of surface plasmon resonance of noble metal nanoparticles [22]. UV–vis absorption spectroscopy is an established method to characterize the formation of silver nanoparticles and their shape [23]. Figure 2 shows the UV–vis absorption spectra of the metallopolymer solution **2a**.

The spectra exhibit absorption in the range of λ_max_ = 450 nm, the typical surface plasmon resonance band of silver nanoparticles. Furthermore the surface plasmon resonance band in this region strongly suggests the formation of spherical particles [24]. With progress of the particle formation the intensity of the plasmon resonance band increases besides a slightly batochromic shift of the band takes place (Figure 2b) according to the increased size of the silver nanoparticles.

**Figure 3:**
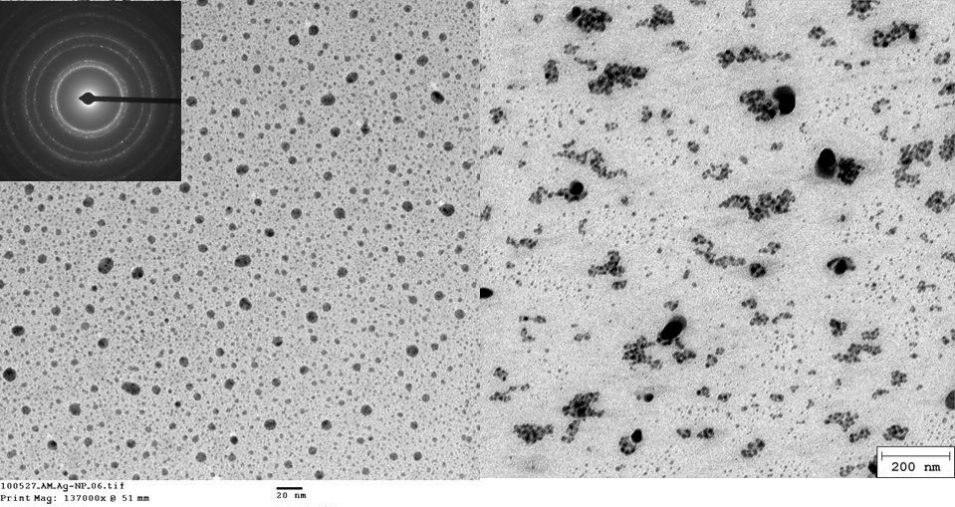
TEM images of polymer stabled AgNP’s **2a** (left) and **2d** (right).

To confirm the postulated spherical shape and to determine the size of the nobel metal nanoparticles transmission electron microscopy (TEM) experiments were carried out. Figure 3 shows two TEM images and corresponding size distributions with different ratios of silver nitrate to cyclodextrin containing polymer **1**. The selected area electron diffraction (SAED) pattern of the Au nanoparticles **2** shows rings ascribed to Ag crystals of the face-centered cubic (fcc) structure (Figure 3, inlet).

**Figure 4:**
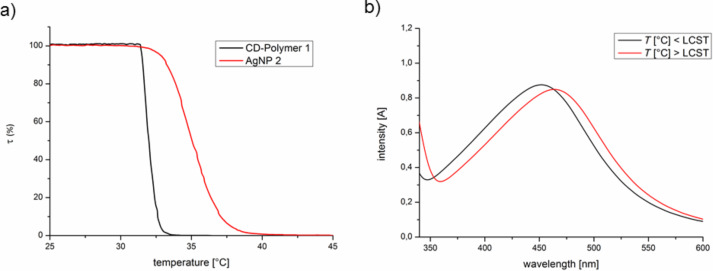
a) Turbity meseaurments of **2a**; b) UV–vis absorption spectra of **2a** below and upon its LCST.

As clearly seen from Figure 4 silver nanoparticles **2** with a spherical morphology were formed. The average size of particles **2a** prepared with 1 mM AgNO_3_ and 10 mM polymer bound CD **1** was determined to be 12.5 ± 2.3 nm with a narrow size distribution. With an increasing molar ratio (1:1) of salt precursor to polymer bound CD **1** an increase of the particle size **2d** up to 100 ± 20 nm and a broader size distribution due to agglomeration is observed. With decreasing polymer concentration the stabilizing capacity is reduced and the polymer cannot restrict the particle size effectively (Table 1).

**Table 1:** Average particle size determined by TEM and DLS.

sample	avg. size [nm] (TEM)	Hydrodynamic diameters *d*_n_ [nm] (DLS)

CD-Polymer **1**	—	7
AgNP **2a**	12.5 ± 2.3	19
AgNP **2b**	31.1 ± 5	19
AgNP **2c**	53.9 ± 12,1	62
AgNP **2d**	100 ± 20	110

Dynamic light scattering experiments were carried out to determine the particle size and the size distribution as function of molar ratio. Hydrodynamic diameters *d*_n_ up to 110 nm prove the formation of silver nanoparticles. Analog to the TEM experiments an increase of the particle size and a broadening of the size distribution with an increasing molar ratio of salt precursor (AgNO_3_) to polymer bound CD **1** due to agglomeration of the AgNP’s (Table 1) was observed. Particle sizes determined by DLS compared to TEM experiments are on average higher due to the fact that in DLS measurements besides the metal particle the polymer shell is measured, too.

Zeta potential experiments were carried out to investigate the stability of the AgNP’s. Measured zeta potentials of the polymer stabled silver nanoparticle solutions **2** show a slightly negative charge (Table 2) indicating a steric stabilization mechanism. The AgNP’s are stable in aqueous medium for weeks. No bathochromic shift of the absorption maximum in the UV–vis absorption spectrum or an increase in the hydrodynamic diameter could be detected and confirm the stability of AgNP’s **2**.

**Table 2:** Experimental conditions of the AgNP’s **2**.

sample	ratio AgNO_3_:CD	conc. AgNO_3_ [mM]	conc. polymer bound CD **1** [mM]

AgNP **2a**	1:10	1	10
AgNP **2b**	1:5	1	5
AgNP **2c**	1:2	1	2
AgNP **2d**	1:1	1	1

Furthermore the optical properties of the polymer stabled nanoparticles were characterized by fluorescence spectroscopy. The fluorescence spectra with a excitation wavelengths λ_ex_ = 225 nm show a emission peakt at λ_max_ = 460 nm in water assigned to fluorescence emission of silver nanoparticles.

Additionally the thermoresponsive optical and solution properties of the polymer stabled silver nanoparticles **2** have been investigated. By heating the AgNP’s **2** above the lower critical solution temperature (LCST) of the NIPAM containing copolymer **1** (33 °C), a volume phase transition will take place, which should effect the optical and solution properties, respectively stability of the silver nanoparticles. As seen in Figure 4a the cloud point *T*_C_ of the native cyclodextrin containing polymer **1** (33 °C) increases up to *T*_C_ = 36 °C with a broadened volume transition due to formation of AgNP’s **2a**.

This shift of the cloud point *T*_C_ is a consequence of the immobilization of polymer **1** on the metal nanoparticle **2** surface due to coordination of silver with the nitrogen atoms of the NIPAM residue respectively hydroxy group of the CD function in **1** [25]. This adsorption suppresses the volume phase transition at higher temperatures [15]. Furthermore the thermoresponsive properties of nanoparticles **2** have been characterized by UV–vis absorption spectroscopy. Figure 4b shows the absorption spectra of a solution of particles **2a** below and upon its LCST. The surface plasmon resonance band λ_max_ = 450 nm at 25 °C is red shifted to λ_max_ = 459 nm when the solution of **2a** is heated about its LCST (40 °C). This bathochromic shift is a consequence of the change of the local environment (decreased distance of Ag particles and increased refractive index of the polymer) of the silver particles **2a** due to the collapse of the polymer [25–27]. The changes in the thermoresponsive as well as the optical properties are fully reversible upon cooling to room temperature.

## Conclusion

In summary, we present the facile one pot synthesis of long time stable silver nanoparticles using a copolymer from *N*-isopropylacrylamide (NIPAM) and mono-(1*H*-triazolylmethyl)-2-methylacryl-β-cyclodextrin acting as reductant and steric stabilizer. We have shown that silver nanoparticles can be prepared under mild conditions without using an external environmental and biological risky reducing agent, stabilizer or additionally energy. Spherical nanoparticles with different sizes have been prepared by altering the molar ratio of silver salt and polymer bound CD. Silver nanoparticles thus prepared are stable over weeks at room temperature and doesn’t appear to aggregate. Furthermore, we were able to show the thermoresponsive reversible changes of solution and optical properties, resulting in bathochromic shift of the surface plasmon resonance band and shift of the LCST to higher temperature.

## Expertimental

### General remarks

All reagents used were commercially available (Sigma-Aldrich, Acros Organics) and used without further purification. β-CD was obtained from Wacker Chemie GmbH, Burghausen, Germany and were used after drying overnight with a vacuum oil pump over P_4_O_10_. *N,N*-Dimethylformamide (DMF) were purchased from Fluka, Germany. Dimethyl sulfoxide-*d*_6_ (99.9 atom % D) was obtained from Deutero GmbH, Germany.

^1^H NMR spectra were recorded on a Bruker Avance DRX 300 at 20 °C, shifts (δ) are given relative to signals caused by the solvent.

FTIR spectra were recorded on a Nicolet 6700 FTIR spectrometer equipped with an ATR unit.

Microwave-assisted synthesis was performed using a CEM Discover Synthesis Unit (monomode system). The temperature was measured by infrared detection maintained at a constant value by power modulation. Reactions were performed in closed vessels.

SEC-MALS measurements were carried out on a combined system comprising the following elements: refractive index detector Optilabrex (Wyatt Technologies, laser wavelength 658 nm), three angle light scattering detector miniDawn TREOS (Wyatt Technologies, laser wavelength 658 nm, detector angles at 43.5°, 90.0° and 136.5°), UV detector Waters 486 (Waters), column set of HEMAbio 300 and HEMAbio 100 (MZ-Analysentechnik), pump, degasser and autosampler (Agilent 1200, Agilent technologies). The eluent was ultrapure water at a flow rate 1 mL/min. The molecular weight was calculated with Astra5 software from static light scattering data, using Zimm-model. As concentration source, the refractive index was used. Calibration of the system was performed with bovin serum albumin.

The absorption spectra were measured on a Specord 210 Plus UV–visible spectrophotometer equipped with a Thermo Scientific CD10 Heating Circulator bath. Fluorescence spectra were recorded on a Perkin Elmer LS55 luminescence spectrometer.

Zeta Potential and dynamic light scattering (DLS) experiments were carried out with a Malvern Zetasizer Nano; ZS ZEN 3600 at a temperature of 20 °C. The particle size distribution is derived from a deconvolution of the measured intensity autocorrelation function of the sample by a general purpose method (non-negative least squares) algorithm included in the DTS software.

Transmission electron microscopy (TEM) images were recorded on a Phillips EM420 (Fa. FEI) microscope at 120 kV. The electron diffraction patternwas recorded for the selected area. The particle size and size distribution was determined by image analysis using AxioVision LE64 software.

Turbidity experiments were performed on a Tepper cloud point photometer TP1. The relative transmission of a laser beam with a wavelength of 670 nm was recorded for each experiment. The measurements were performed at a temperature range between 10 and 40 °C and a heating rate of 1 °C min^−1^ using Hellma Suprasil precision cells 110 Q-S. Critical solution temperatures derived from these experiments were determined at 50% relative transmission.

### Synthesis of mono-(6-azido-6-deoxy)-β-CD

Mono-(6-azido-6-desoxy)-β-cyclodextrin was synthesized according to the known procedure [28].

### Synthesis of and mono-(1*H*-triazolylmethyl)-2-methylacryl-β-cyclodextrin

Mono-(1*H*-triazolylmethyl)-2-methylacryl-β-cyclodextrin was synthesized according to the known procedure but without the addition of copper(II) sulfate pentahydrate and sodium ascorbate and the reaction time was elongated to 60 min [28].

### Synthesis of poly(*N*-isopropylacrylamide-co-mono-β-CD-methacrylate) (**1**)

Poly(*N*-isopropylacrylamide-co-mono-β-CD-methacrylate) was synthesized according to the known procedure [20]. *M*_n_ = 20,000 g/mol determined by SEC; D = 3.2; CD-monomer/NIPAM = 1:10 determined by ^1^H NMR.

### Synthesis of silver nanoparticles (**2**)

For the synthesis of silver nanoparticles (**2**) AgNO_3_ and poly(*N*-isopropylacrylamide-co-mono-β-CD-methacrylate) (**1**) in different molar ratios (see Table 2) were dissolved in 5 mL ultrapure water under pH neutral conditions at room temperature and shaken for 15 min. The color of the polymer solution changed during the reaction from colorless, over orange to brown confirming the formation of the silver nanoparticles **2**.
